# The Multiscale Systems Immunology project: software for cell-based immunological simulation

**DOI:** 10.1186/1751-0473-3-6

**Published:** 2008-04-28

**Authors:** Faheem Mitha, Timothy A Lucas, Feng Feng, Thomas B Kepler, Cliburn Chan

**Affiliations:** 1 Center for Computational Immunology, Department of Biostatistics & Bioinformatics, Duke University Medical Center, 2424 Erwin Road, Hock Plaza Suite G06, Durham NC 27705, USA; 2 Department of Immunology, and Department of Statistical Science, Duke University, Durham NC 27705, USA

## Abstract

**Background:**

Computer simulations are of increasing importance in modeling biological phenomena. Their purpose is to predict behavior and guide future experiments. The aim of this project is to model the early immune response to vaccination by an agent based immune response simulation that incorporates realistic biophysics and intracellular dynamics, and which is sufficiently flexible to accurately model the multi-scale nature and complexity of the immune system, while maintaining the high performance critical to scientific computing.

**Results:**

The Multiscale Systems Immunology (MSI) simulation framework is an object-oriented, modular simulation framework written in C++ and Python. The software implements a modular design that allows for flexible configuration of components and initialization of parameters, thus allowing simulations to be run that model processes occurring over different temporal and spatial scales.

**Conclusion:**

MSI addresses the need for a flexible and high-performing agent based model of the immune system.

## Background

Computer simulations are becoming increasingly important in biological research, complementing both laboratory experiments and the venerable models of mathematical biology. While the line between mathematical modeling and simulation is somewhat indistinct, simulation typically incorporates a greater level of biological detail than mathematical models, facilitating the mapping between the biological and formal representations at the cost of increased complexity and reduced analytical tractability. This more direct correspondence allows subject-matter specialists with little mathematical experience to participate in the design and interpretation of computational experiments more easily; a consideration of great importance for the evolution and improvement of the model.

We have undertaken the development of an individual cell-based simulation system comprising components of the immune system, based on the relevant biophysics and intracellular dynamics, to help elucidate the early immune response to vaccination and natural infection. We believe that such a framework for immunological simulations can be used to explore the details of molecular and cellular dynamics, in much the same way as a new optical technology such as two-photon microscopy has radically advanced our understanding of leukocyte dynamics by permitting visualization of *in vivo *cell motility and interactions. The construction of realistic complex spatiotemporal simulations, however, is relatively new to mathematical and theoretical immunology, and poses significant software engineering challenges with which few researchers in the field will be familiar.

The immune response to an antigenic challenge such as vaccination consists of processes occurring over several temporal and spatial scales, from intracellular signaling (seconds to minutes), to the complex spatial reorganization that occurs in the draining lymph nodes some distance away (hours to days). The outcome depends on interactions between several cell types communicating by direct cell-cell contact and over short distances by diffusible small messenger molecules known as cytokines, and on the interactions of the motile immune cells, known as leukocytes, with the resident parenchymal cells. Adding to the complexity, all these interactions may be modulated by the local extracellular matrix, which guide the motions of leukocytes and provide a substrate upon which bound cytokines interact with these cells. While many of these individual interactions have been studied in detail experimentally, there is yet little real understanding of how these components add up to produce a coherent immune response. The reconstructive approach embodied by computational simulation holds promise for effecting the necessary synthesis.

We therefore describe the challenges and trade-offs inherent in building such a simulation, and our specific choices, in the hope that other researchers will gain a better understanding of the issues involved and consequently make more informed software engineering choices. Finally, we compare our package with two closely related simulation software packages with similar goals, namely Compucell [1-3] and Simmune [4,5], that illustrate complementary approaches to engineering complex immune simulation software.

## Implementation

### Development Environment

The first decision was that of programming language. We wanted a design driven by the immunological domain we are simulating, while maintaining the high performance necessary for such a complex simulation, which would have to deal with reaction-diffusion equations as well as the intracellular dynamics and interactions of thousands to millions of leukocytes and parenchymal cells. Our specific needs for a language included support for robust pseudorandom number libraries, multi-dimensional arrays and parallel computation. While languages such as Fortran and C fit these requirements well, they failed at our other requirement of a flexible high-level interface for description of the model domain. C++ includes C as a subset while providing high-level object-oriented facilities, and also has the advantage of being well-known and familiar to the scientific community. For better or worse, C++ has strong static typing, and this basic feature to a large extent dictates usage of the language. Strongly typed languages are comparatively inflexible and are poorly suited to rapid development, particularly in exploratory modes of programming. As a compromise, we opted to use a mixed language environment, where an interpreted dynamically typed language extends a statically typed compiled language. This hybrid system to some extent inherits the advantages of both languages.

For pragmatic reasons, we decided on a Python/C++ hybrid. Python is a popular object-oriented interpreted language in widespread use as a scripting language, but it also has good and ever-improving scientific support. It is also well-suited as a glue language, facilitating the connection of the base simulation code with databases and visualization systems. An important technical tool that made such a hybrid system feasible was the existence of the Boost Python library [6], which offers a sophisticated C++ API to convert functions and data types automatically to and from the two languages. One approach to exploiting such a hybrid system is to code most of the simulation in Python, using C++ only for the time-intensive routines. We were constrained, however, by the need for high performance and the consequent need to develop for implementation on Beowulf cluster architectures; the Python MPI wrappers are, for this purpose, unacceptably slow. We chose instead to create two functionally identical systems, one in Python and one in C++. Synchrony between the two was enforced by writing a suite of Python unit tests, which depending on a switch, tests either the Python directly or C++ system via Boost Python wrappers. This seems to enhance productivity: it is simpler to code and test prototypes in Python and, at the appropriate point in their development, port them to C++ than to develop and test prototypes from scratch in C++. We also take advantage of flexible and efficient arrays in Fortran by calling individual Fortran routines from C++ for low level matrix and vector computations.

Just as fundamental were the choices we made for our software development environment. We chose to use the distributed version control system Mercurial [7], because a distributed version control system offered superior support for multiple developers in terms of branching, merging and disconnected operations. For software builds, we chose Scons [8]. Among other benefits, Scons automatically manages file dependencies, and allows us to customize our build scripts using the full power of Python. We elected to use Trac for bug and issue management, as it allows us to manage version control, bug tracking and documentation within the same web-based package. Not coincidentally, all the tools we chose are Python based, which makes it possible for us to extend these tools if necessary.

### Design

One of our design constraints was that the primary domain for our simulation was molecular and cellular immunology, not mathematics, physics or computer science. At the least, this meant that we had to be able to specify and describe a simulation run using the vocabulary of immunology. Another constraint was that we were constantly researching new prototypes, adding more functionality to the system and investigating more efficient data structures and algorithms for performance bottlenecks, so the system had to be extensible; ideally, we would be able to swap in new functionality for old with minimal additional effort. To meet these constraints, we decided on a hierarchical modular system. The implementation of such a system was naturally object-oriented, a paradigm both C++ and Python support. At the top level, we split the project into Catalog, Core and Graphics/Analysis modules. Within each of these were separate sub-modules and classes that actually implemented the functionality of the system. This organization allowed us to develop individual modules independently, so long as there was a standard API that allowed communication between modules.

The Catalog module is a repository of biological and physical information. Its entries include, for example, parameters governing the locomotion and behavior of specific leukocyte types and the diffusion coefficients and reaction rates of cytokines. The information in the Catalog is stored in a PostgreSQL relational database, and simulation classes are mapped to database tables using the strategy of mapping each inheritance tree to a table. To facilitate data entry into the Catalog, the Python library

SQLAlchemy citesqlalchemy was used to provide an object-relational mapping, making it possible for developers to create new class specifications without knowing SQL. Our plans call for the development of a web interface to the Catalog, so that experimentalists and other non-developers can contribute items to the database. After describing the Experiment module, we will describe how the Catalog is used to construct class entities in a simulation at run-time.

**Figure 1:**
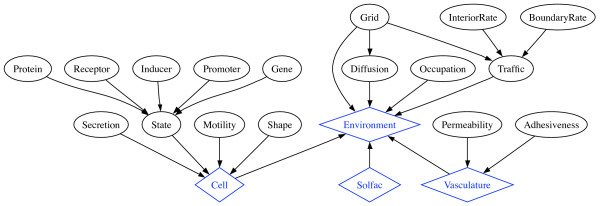
**Class composites and aggregates in the Core module**. Top level classes (Environment, Vasculature, Cell, Solfac) are shown as diamonds and component classes that provide specific functionality as ovals. Instances of component classes are passed in as parameters to the constructor of its enclosing class for a flexible specification of the behavior of the top level classes. The Environment instance contains cell, soluble factor and vasculature instances which are autonomous and may interact with each other.

The Core module is where the code for running a simulation actually resides. To allow the description of a computational experiment using immunological terminology, there are only a few base classes in the public interface that a typical simulation is required to specify, namely Simulation, Environment, Cell, Vessel and Soluble_factor (Figure 1). We have designed our classes to have a relatively flat inheritance structure and to use abstract base classes where possible. We find that this design promotes loose coupling and facilitates maintenance.

The Simulation class manages administrative tasks, with the ability to run or step through a simulation and log simulation details. It will also provide hooks to interface with run-time logging, computational steering and visualization systems when these are developed.

The Environment class represents a physical spatial volume, which may be simple, such as when modeling an *in vitro *environment, or complex, such as when modeling physiological tissue. Since our task allocation is done in a parallel environment using a spatial decomposition, each processor in a parallel run will contain its own Environment instance. Most of the work done by the Environment class is delegated to three private classes which handle the reaction-diffusion equations (Diffusion), collision detection (Occupation) and cellular immigration and emigration (Trafic). Since these classes are an implementation of the well-known Strategy design pattern, it is simple to upgrade the functionality provided by simply pointing to a new class. For example, we recently replaced the original forward Euler scheme with a Multigrid reaction-diffusion algorithm [9], resulting in an order of magnitude speedup. This was accomplished by simply replacing the Diffusion class in Environment. The Environment also serves as a container for various cell types (Cell), blood and lymphatic vessels (Vessel), and various cytokines and chemokines (Soluble_factor), and manages the interactions among these components.

Similar to Environment, the Cell base class is also a composite class, with distinct behaviors delegated to their own classes, and hence also extensible. A cell's functional state is given by, among other things, the local concentrations of various proteins in each of their post-translationally modified states (*e.g.*, phosphorylated on specific residues). These characteristics of a protein are themselves dynamically regulated, and typically modeled using nonlinear ordinary differential equations. Importantly, these proteins determine the functional behavior of the cell, and it is sufficient to couple cell behavior modules to these protein concentrations to model cell stimulus-response behavior. The protein readouts are encapsulated in a State class, which tracks and exposes the fluctuating protein levels. In our current implementation, there is a mechanistic model that governs the protein levels, in which the rates of protein synthesis and/or degradation are governed by an explicit cascade involving model classes for Receptors, Inducers, Promoters, Genes and regulatory Proteins. It would be straightforward, however, to replace this mechanistic model with a probabilistic model, or even a much more detailed mechanistic model depending on the goals of the simulation. By having a flexible granularity, we can study an immune response at different levels of spatiotemporal resolution. When studying the behavior of individual cells, for example, we can increase the resolving power, while for studying large population behaviors, a coarser approximation of internal cell dynamics might be sufficient. Other behaviors like Motility and Secretion can then base the quality/quantity of their response at any time step on the internal environment represented by the State class and/or the external environment (e.g. concentration or gradient of various cytokines or cell-cell contacts). This design allows an extremely flexible stimulus-response coupling between the internal and external cellular environments and cellular behavior. Because of the loose coupling and the ability to change cellular behavior by "plug-in", an inheritance hierarchy to represent the different cell types is not necessary. Instead, we just "plug-in" different behavior modules for the various cell types to implement their observed behaviors. In practice, this means that specific behaviors for a particular cell type are implemented as instances of their respective classes that are given to the cell class constructor as parameters.

The Vessel class is used for the representation of the blood vessels that permeate host tissue. Leukocytes typically enter tissue by migrating across the endothelial barrier at capillaries, and egress at post-capillary venules to enter the lymphatic system. The rate of entry and egress of various cell types is regulated by pro-inflammatory cytokines, which regulate the adhesiveness and permeability of the blood vessels, as well as by chemokines, which influence leukocyte migratory properties. Our implementation of blood vessels is simple; the entry and egress vessels are stored in matrices representing positions in space, and the probability of cell trafficking at a point depends on the permeability and adhesiveness of the corresponding matrix entry, which in turn is sensitive to the local concentration of the relevant soluble factors. We currently assume that the blood vessels are homogeneously distributed; it is trivial to relax this assumption to reflect tissue histology more accurately by masking certain matrix entries.

The Soluble_factor class is essentially a container for a array of concentrations, with a few accessor and mutator methods for convenience and some basic attributes including its diffusion coefficient. An instantiation of the Soluble_factor class is made for every cytokine, chemokine and other soluble factor of concern in the simulation. The array is updated by the Diffusion class which takes into account secretion from cellular sources, adsorption by cells and extracellular matrix, binding reactions between soluble factors, and diffusion. There are several classes derived from Diffusion that represent different methods for solving the diffusion problem, including a forward Euler method and a backward Euler method which incorporates multigrid.

While the bundling of distinct behaviors into separate classes certainly simplifies the dynamical configuration of classes, their instantiation can be clumsy, accounting for a significant proportion of the code required to run a simulation. Biological cells, however once adequately characterized, can be reused in many different simulations, just as an experimentalist would use the same cultured cell lines (possibly ordered from a catalog!) for many different experiments. This was the idea driving the creation of the Catalog module, and here we will describe how it is used for instantiation of Experiment classes.

We started by writing generic code that could construct a class instance without advance specification of the instance type or even its constructor parameters. This design provided the flexibility to easily incorporate new classes in the database, or new subclasses of currently existing types. To do so, we adopted the object factories scheme, which allows flexible creation of object instances using C++ templates [10].

**Figure 2:**
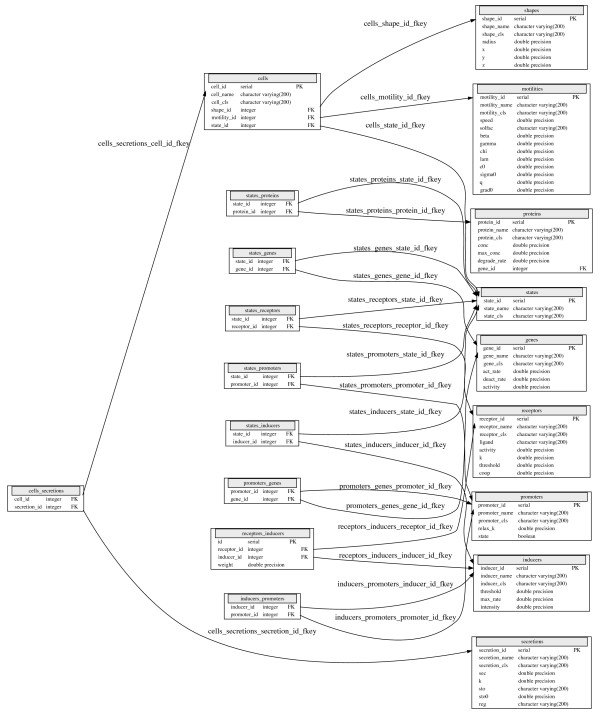
Database schema for the Cell class in the Catalog.

We also extended the factory to take arbitrary constructor parameters with partial template specialization, and wrote a Python script to generate templates for up to 20 arbitrary constructor arguments. Our specific implementation of the factory pattern follows [11]. To create a factory for a particular class, we instantiate the factory and register it with the appropriate constructor arguments passed in as a string. While this process is a somewhat involved, it has to be done only once for each class; the typical user does not have to be exposed to any of it, since all that is required once the infrastructure is in place is to call a function such as create_cell("macrophage"), which will call the appropriate constructor for a macrophage. As described earlier, a cell such as a macrophage is a hierarchically organized composite class, and each subclass in the hierarchy will require the appropriate parameters to be passed in to its factory. We store the parameters for instances of each class in the open source relational database PostgreSQL, using foreign key constraints to relate the correct subclass instances in any given object hierarchy (Figure 2). Foreign key constraints allow one field in a database table to refer to fields in another table, allowing information retrieval from tables with a matching entry. The component parameters can then be retrieved via such foreign key references when a specific cell type like macrophage is constructed, and this ensures that the appropriate parameters are used to instantiate all levels of the composite class. In practice, we connect to a local or remote database containing the data using the libpqxx C++ API for PostgreSQL at the beginning of the simulation, and also use the libpqxx utility functions to execute the necessary SQL statements to find the correct parameters when the constructor is called.

For visualization and analysis, we export simulation variables using the standard HDF5 format [12], which can then be visualized using our own wxPython and OpenGL based visualization application or imported into a statistical package such as R or Matlab for further analysis.

### Mathematical Considerations

The standard equations that describe the dynamics of the soluble factors as well as the cells are as follows. Suppose *c*_*i*_(**x**, *t*) is the local concentration of soluble factor *i *at the position **x **and time *t*. Let **x**_*μ *_be the position of the center of cell *μ *in the bounded domain Ω ∈ ${\mathbb{R}}^{3}$. Then the reaction-diffusion equations that describe the behavior of the soluble factors from [13,14] are

(1) $$\begin{array}{lll} \frac{\partial c_{i}}{\partial t}(x,t) & = & \left\{D_{i}\Delta -\sum_{j=1}^{N}r_{ij}c_{j}(x,t)-\lambda_{i}\right\}c_{i}(x,t)\\ & & \begin{array}{cc} +\sum_{\mu =1}^{M}J_{i\mu }(x,t)g_{\mu }(x-x_{\mu }(t)), & x\in \Omega . \end{array} \end{array}$$

where *g_μ _*is a smoothly cut-off Gaussian with support over the volume of the cell. Here *D*_*i *_denotes the diffusion coefficient for soluble factor *i*, *r*_*ij *_is the rate at which soluble factor *i *is removed by interaction with soluble factor *j*, and *λ*_*i *_is the rate of removal of soluble factor *i *by other processes. These positive constants are stored in the Soluble_factor class. The secretion of soluble factor *i *through the surface of cell *μ *is approximated by a source term centered at the cell position **x**_*μ *_with secretion rate *J_iμ_*(**x**, *t*). Although the model in [13,14] contains point sources, the existence of a solution to (1) requires a smooth source term as discussed in [9]. In the simulation, the rate *J_iμ _*(**x**, *t*) is determined by the Secretion member of each cell. The source term for each soluble factor is a sum over the *M *individual cells indexed by *μ*.

The system (1) is coupled with *M *systems of stochastic differential equations that describe the motions of each cell individually. For each cell, indexed by *μ*, denote by **x**_*t *_∈ ${\mathbb{R}}^{3}$ the position and by $v_{t}\in \mathbb{R}$ the velocity. The cell motion is modeled by the Langevin process

(2) $$\begin{array}{lll} dx_{t} & = & \begin{array}{cc} v_{t}dt, & x(0)=x_{0}, \end{array}\\ dv_{t} & = & [h(c(x_{t}))-\gamma v_{t}]dt\\ & & \begin{array}{cc} +\sigma (c(x_{t}))\sqrt{\gamma }dW_{t}, & v(0)=v_{0} \end{array} \end{array}$$

where *dW*_*t *_is a standard Wiener process in ${\mathbb{R}}^{3}$. The cell velocity is stochastic but biased toward the direction of the gradients of the relevant soluble factors by the relation

(3) $$h(c)=\sum_{i=1}^{N}\frac{\chi_{i}\nabla c_{i}}{h_{0}+|\nabla c_{i}|}.$$

Here *χ*_*i *_is the chemotactic constant that controls how much the drift is influenced by the gradient of soluble factor *i *and *h*_0 _is the length of the gradient of *i *at which *h *attains half its maximum value. The magnitude of the Wiener process depends on the soluble factor concentration by

(4) $$\sigma (c)=\sigma_{0}{\left(1+\frac{c}{c_{0}}\right)}^{q}e^{-\lambda c}.$$

The positive constants *γ*, *c*_0_, *h*_0_, *σ*_0_, *q *and *χ*_i _are stored in the Motility class, which is a member of Cell.

The reaction-diffusion-stochastic system (1), (2) contains a set of coupled partial differential equations that interacts with a set of stochastic differential equations. Instead of solving this complex system, we consider the following three problems for which there are known numerical methods:

1. The diffusion of the soluble factors,

(5) $$\begin{array}{c} \frac{\partial c_{i}}{\partial t}(x,t)=D_{i}\Delta c_{i}(x,t)\sum_{\mu =1}^{n}J_{i\mu }(x,0)g_{\mu }(x-x_{\mu }(0)),\\ c(0)=c_{0}(x). \end{array}$$

2. The reactions of the soluble factors,

(6) $$\begin{array}{c} \frac{\partial c_{i}}{\partial t}(x,t)=-\left\{\sum_{j=1}^{m}r_{ij}c_{j}(x,t)+\lambda_{i}\right\}c_{i}(x,t),\\ c(0)=c_{0}(x). \end{array}$$

3. The motion of the cells (2).

Given initial concentrations *c*_0_(**x**), initial positions **x**_0 _and initial velocities **v**_0_, we can approximate *c*_*i*_(**x**, Δ*t*), **x**(Δ*t*) and **v**(Δ*t*) by the following splitting scheme [9]:

1. Solve the diffusion, (5), for Δ*t *using the initial data *u*_*i*_(**x**, *t*), and **x**_0 _and **v**_0_.

2. Solve the reaction, (6), for Δ*t *using the solution from the previous step as initial data.

3. Move the cells according to (2) for one time step Δ*t *using the soluble factors from the previous step and the initial cell positions and velocities, **x**_0 _and **v**_0_, as initial data.

The error due to this splitting is O(Δ*t*). This result is well-known for ordinary and partial differential equations; we have extended it to the reaction-diffusion-stochastic system [9]. Furthermore, if the numerical schemes for (5), (6) and (2) are at least O(Δ*t*), then the resulting error is O(Δ*t*). Here we see that operator splitting allows us to solve three tractable problems rather than one complex system. Splitting the reaction and the diffusion reduces a system of nonlinear partial differential equations to a system of linear partial differential equations for the diffusion, and a system of ordinary differential equations for the reaction. The partial differential equations can be solved using a backward Euler scheme with multigrid for the linear solve as described in [15] and [16]. The multigrid scheme is implemented as a member function of the Multigrid_diffusion class which is derived from Diffusion. This class contains a necessary set of parameters, as well as functions to compute the relevant sinks and sources from the list of cells. The reaction equations are solved numerically using a first order semi-implicit Euler scheme. A separate scheme for the reaction is also a member function of the Diffusion class; it updates the entire list of soluble factors for one time step. Finally, the Langevin process for the cell motion can be simulated exactly as described in [17]. The Langevin motility class stores a set of parameters that are relevant to the equations and numerical scheme. It also contains member functions that use the concentrations of the soluble factors to update the velocity and return the displacement of the cell for one time step. Although the soluble factors and the cells depend on each other, operator splitting allows us to update them sequentially instead of simultaneously and further modularizes the simulation.

**Figure 3:**
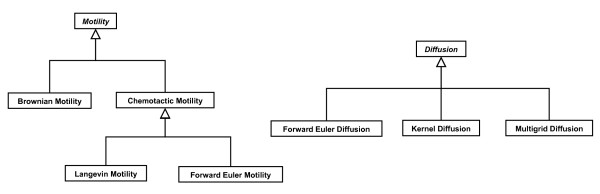
Motility and Diffusion inheritance trees.

We have also implemented multiple schemes for diffusion, reaction and cell motility, which are derived classes from Diffusion and Motility (Figure 3). The derived classes may represent a change in the dynamical equations themselves or in the numerical methods used to solve them. For each simulation, the user can choose a set of equations and schemes that best models the desired behavior, approximates the mathematical equations most accurately or produces results most efficiently. In particular, different cell types can display different motility behaviors within the same simulation. As researchers develop more realistic models and more accurate and efficient numerical methods, the system facilitates simple swapping of these new classes for old ones and simplifies comparison and testing.

### Testing

We run unit tests using the Python unittest module, with the only novelty being that since all our C++ classes are compiled to a shared library readable in Python, we can drive both Python and C++ unit tests with the same test code. At a slightly higher level, the user may compare simulated behavior with analytical predictions where closed form solutions (either deterministic or statistical) are available. Examples include testing cell motility modeled as Langevin processes or simple diffusion from a single point-source secreting at a constant rate. There are also informal eyeball tests, in which simulations with simple configurations are run and visually checked to ensure that the resulting behavior is qualitatively consistent with experimental results. We have not incorporated code coverage analysis [18] or continuous build systems [19] yet, but these would certainly be sensible additions.

### Optimization

Due to the highly modular nature of our code, it is generally simple to replace an inefficient module with a more efficient one. Identification of bottlenecks is done using the *callgrind *tool widely available on Unix platforms, and the *kcachegrind *GUI to visualize the resulting profile. Examples of such high level optimizations include the replacement of a forward Euler diffusion scheme with a Multigrid scheme, and replacing collision detection by looping over all cells with a grid-based method implemented as a hash table. At a lower level, we have removed unnecessary copies by using references where possible for large data structures, and rearranged code to minimize unnecessary looping. So far we have avoided doing micro-optimizations (e.g. loop unrolling) believing that such tasks are best left to the compiler. One simple optimization we did incorporate was to specify the appropriate compiler flags for each machine architecture, allowing the compiler to make optimal use of pipelining and vectorization.

## 1 Results and Discussion

### Functionality

Running a complex simulation inevitably requires a fairly sophisticated supporting computer software infrastructure. The primary purpose of the MSI software is to provide such an infrastructure for running simulations of the immune response, making it possible for users inexperienced in simulation methods to conduct, visualize and analyze complex computational experiments. The functionality provided by the software falls into three main categories – providing plug-in components for an immune simulation that can be assembled to form complex simulations, data and parameter management via a relational database, and a graphical user interface for visualization and control.

The MSI software provides components that model both the *physical *and *biological *aspects of an immune simulation as a hierarchy of loosely coupled classes. We provide physics-based classes that model chemical reaction and diffusion, contact forces and collisions, and biology-based classes that model the environment, cells and soluble factors. Some of these may be composite classes and have sub-components that can be plugged in to provide new functionality. Cells can, for example, be fitted with different model classes that provide specific motility, secretion, sensory abilities etc.

The development of useful biological models requires extensive parameterization, typically done by mining the available literature or conducting experimental measurements. Within the MSI system, these data are managed by storing the parameters that characterize specific biological entities (e.g. cell types such as macrophages, neutrophils, fibroblasts, etc.) in a relational database. The database is closely integrated with the model classes in the code, so that biological entities can be instantiated by the appropriate database name lookup. The integration of the database catalog of biological entities and model classes makes it possible to specify a computational experiment using the appropriate domain by simply specifying the appropriate environment, cell types and soluble factors that must be present in the simulation. Clearly, this framework also makes re-use simple; the same cell types used in one simulation can be used to populate another one with, say, a different environment.

Another aspect of data management is the collection of the vast quantities of numerical and descriptive data that can be generated in simulation runs. To support this functionality, we use the HDF5 hierarchical data format developed by NCSA, which is highly efficient, scalable and provides a utility library for data access and manipulation. The HDF5 files are stored for off-line analysis and visualization. Paraview [20] is an example of a tool that can read and parse the stored HDF5 files to generate an openGL-based rendering of the simulation.

**Figure 4:**
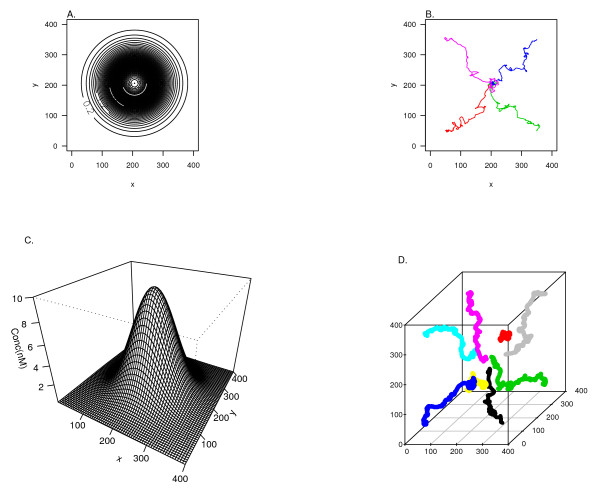
**Behavior of cell modules in a point source chemotactic field in two and three dimensions**. (A) The chemokine concentration in a 2-D plan (400 mm by 400 mm) following a Gaussian distribution. (B) The trajectories of 4 cell modules, each starting from one of the corners of this 2-D plan. (C) The concentration profile of chemokine of the middle section through the 3-D tissue volume. (D) The trajectories of 8 cell modules starting from the corners of the 3-D tissue. This simple simulation of cell chemotaxis involves the interaction between the Motility (as part of Cell), Soluble factor and Diffusion (as part of Environment) classes in the system. (B) and (D) were generated by simply changing the "dim" template argument, as an example of the generic programming abilities afforded by the C++ language and built into the system.

The primary purpose of the MSI software is to run large-scale cell-based immune simulations. A basic example of individual cellular motile behavior governed by Equations (2), (3) and (4) where leukocytes migrate up a fixed chemotactic gradient in both 2D and 3D is shown in Figure 4. To show how the software can be set up to run such simulations, we provide a pseudocode walk-through of a slightly more complex simulation in which three discrete point sources set up dynamic soluble factor gradients, which in turn recruit and activate leukocytes.

**Figure 5:**
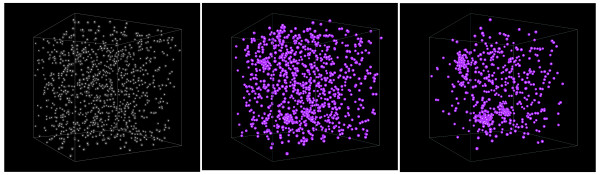
**Snapshots of test_simulation as described in Table 1**. Snapshots are shown at 0 (left), 5 (center) and 10 (right) hours of simulation time. Cell colors indicate degree of activation of pro- and anti-inflammatory genes.

The first step in the simulation is initialization of the database, in which the parameters of the various objects are stored. This results in the creation of a singleton DBMaker instance, which provides the factory functions to create all the simulation classes by name. An environment is created and populated with cells serving as point sources, three soluble factors and a vasculature that regulates cell immigration. Finally, a specified number of macrophages is initially created and put in random positions. The simulation itself consists simply of a high level call to step through time in fixed increments *dt*, which in turn updates the cells, soluble factors and vasculature. Each time step is then logged to an appropriate data storage format like HDF5. Snapshots of this simulation are shown in Figure 5, and pseudocode is given in Table 1.

**Table 1:** test_simulation pseudocode

**procedure **Initialize(*n*_0_)	▹ Number of leukocytes.
DBConnStr ← (user, dbname, host, port, password)	▹ Initialize database.
DBMaker ← DBMaker::Instance(DBConnStr)	
Environment ← DBMaker.CreateEnvironment("testenv")	
Source1 ← DBMaker.CreateCell("source")	▹ Creating and adding point sources to Environment.
Source1.SetPosition(*x*_1_, *y*_1_, *z*_1_)	
Environment.AddProp(Source1)	
Source2 ← DBMaker.CreateCell("source")	
Source2.SetPosition(*x*_2_, *y*_2_, *z*_2_)	
Environment.AddProp(Source2)	
Source3 ← DBMaker.CreateCell("source")	
Source3.SetPosition(*x*_3_, *y*_3_, *z*_3_)	
Environment.AddProp(Source3)	
TNF ← DBMaker.CreateSolfac("tnf")	▹ Creating and adding soluble factors to environment.
Environment.AddSolfac(TNF)	
STNFR ← DBMaker.CreateSolfac("stnfr")	
Environment.AddSolfac(TNF)	
MCP1 ← DBMaker.CreateSolfac("mcp1")	
Environment.AddSolfac(TNF)	
Vasculature ← DBMaker.CreateVasculature("testvessel")	
Environment.AddVasculature(Vasculature)	
**for ***i *← 1, *n*_0 _**do**	▹ Creating and adding leukocytes to environment.
Cell ← DBMaker.CreateCell("macrophage")	
Cell.SetPosition(Random(Environment.Bounds))	
Environment.AddCell(Cell)	
**end for**	
**end procedure**	
**procedure **Main(dt, numsteps)	
Initialize(*n*_0_)	
Simulation ← new Simulation(Environment)	
**for ***i *← 1, numsteps **do**	
Simulation.Step(dt)	
Log(*i*, Simulation)	▹ Log of simulation results.
**end for**	
**end procedure**	

We have also recently completed a series of computational experiments which illustrate how the software can be used to gain insight into a biologically relevant process – the mechanisms regulating inflammation following a tissue insult. The insult triggers the release of chemotactic soluble factors (chemokines) by parenchymal cells (fibroblasts), which in turn recruit immune cells (leukocytes). Using multiple simulation runs to systematically perturb the system variables, we studied the feedback regulation of tumor necrosis factor alpha (TNF-*α*) by its shed receptor soluble TNF receptor (sTNFR) and its effect on the recruitment and spatial distribution of the leukocytes over time. The results have been published [14] and serve to illustrate the kinds of simulations enabled by the MSI software package.

The two most closely related software packages that simulate multiple cells interacting in a dynamic environment for immune simulation are Compucell [1-3] and Simmune [4,5]. Compucell uses the cellular Potts model to model cell dynamics by partitioning space into pixels, each of which is regarded as a cellular automata. The overall behavior of individual cells, modeled as a collection of pixels, depends on minimizing the total effective energy of the system subject to constraints that channel the dynamics. In contrast to our direct mapping of cell behavior modules to the underlying biology, the specification of cell behaviors in Compucell is indirect, via constraint terms in the energy function. Such an abstract specification limits its appeal to experimental immunologists, since it is difficult to translate the simulation to detailed biological mechanisms. Simmune is also an agent-based model with a sophisticated user interface design that allows a graphical specification of the simulation biochemistry and inter-cellular interactions by biologists. In particular, Simmune uses a GUI which allows cellular behavior to be copied across cell types. Also, it simulates explicit reaction-diffusion across all scales, and uses an adaptive integrator to separate the different timescales with a user-defined precision. We agree with Simmune's philosophy that immune simulations must have models that map closely to the underlying biology to be useful to experimentalists. Our approach is slightly different, in that we provide 'default' collections of cells and cellular behaviors in the Catalog, that can be used as a template and easily modified to simulate experimental perturbations, making it simpler to set up typical simulated experiments of cellular immune responses. Currently, the software emphasis is also different – Simmune focuses more on biochemistry, while we have paid more attention to the biophysics of cell motility and diffusion from a motile source. Due to the complexity and scope of immune systems, we strongly believe that multiple complementary approaches to large-scale quantitative immune simulations will continue to develop and cross-fertilize each other's development.

### Roadmap

The code is currently functional and has been tested on Debian, Ubuntu, OpenSuSE and Mac OS X platforms. We are hereby releasing it as an Open Source project and invite interested developers to participate in the project. Source code, documentation and a community wiki can be found at the Multiscale Systems Immunology website [21]. In the near future, our priorities are to populate the Catalog with additional cell types, soluble factors and environments, so that more realistic and complex immune simulations can be conducted. We also plan to fully parallelize the code using MPI so that larger-scale simulations can be run in a reasonable time on a moderate-size Beowulf cluster. We are also prototyping more capable visualization frontends based on the Visualization Toolkit (VTK) [22], as well as the possibility of real-time visualization and control with computational steering techniques. We intend to provide the means to simulate immune processes in heterogeneous environments with virtual tissue architectures based on histological analysis of biological tissues. The primary challenge for this task will be the development of efficient methods for the integration of reaction-diffusion equations in irregularly bounded spaces. Our group is actively engaged in research in this area. Finally, while we use the software exclusively for simulating immune responses, the software is sufficiently generic to run simulations of any cell population phenomena with minimal adaptation.

## Conclusion

There are many software engineering techniques that can enhance productivity and make more pleasant (or at least less painful) the development of large, complex scientific simulations. Since most biological modelers today come from a more theoretical background and their coding experience is often limited to smaller single-developer efforts, these important techniques may not be familiar to researchers who could most profit from them. We have here described some of the tools and methods that we have found useful in the construction of an cell-based immune simulation, including setting up sensible version control and build systems, and testing suites; the use and integration of a relational database, modular decomposition of the project, object-oriented design incorporating ideas from domain-driven design and the patterns community, and the use of standard formats to facilitate communication between subsystems (RDBMS, HDF5). Perhaps more unusually, we have opted to use a mixed language approach to software development – this choice has worked well for us, and we are comfortable recommending it to others. We hope that this paper will help other groups develop better, more sophisticated simulators for molecular and cellular biology, and allow a more integrated understanding of organisms.

## Availability and requirements

• Project name: The Multiscale Systems Immunology (MSI) Project.

• Project home page: [21].

An apt repository for the MSI code is available for readers using the Debian or Ubuntu operating systems. This repository currently contains the source code and binary packages for Debian etch (i386 and amd64) and Ubuntu feisty (i386 and amd64). The source code can also be obtained here as a tar.gz file [Additional file 1]. For further information about the apt repository, see the README at [21] (Documentation → Installation from the main MSI project page).

• Operating system(s): Linux (tested on Debian 4.0 (etch) and Ubuntu 7.04 (Feisty Fawn)).

• Programming language: Python, C++ and Fortran.

• Other requirements: See README in source for code dependencies.

• License: GPL.

## List of abbreviations

API: Application Programming Interface; HDF5: Hierarchical Data Format 5; IDE: Integrated Development Environment; MPI: Message Passing Interface; NCSA: National center for Supercomputing Applications; PDE: Partial Differential Equation; RDBMS: Relational Database Management System; SQL: Structured Query Language; VTK: Visualization ToolKit.

## Authors' contributions

FM, CC and TBK co-wrote the manuscript, designed the object-oriented architecture, and implemented the bulk of the Python and C++ code. FM selected and implemented the software infrastructure for the project. CC designed and implemented the visualization and database modules. TAL researched and implemented numerical methods for the partial and stochastic differential equations, contributed to the overall program design and drafted the *Mathematical Considerations *section of the manuscript. FF fitted the model to the experimental data, generated the parameters for the cell motility module, did unit and systemic tests of the system, and generated figures for the manuscript. TBK conceived the project, built the first prototype, developed the biophysical models, and secured its funding. All authors read and approved the final manuscript.

## Supplementary Material

Additional file 1 **MSI source code**. Source code for the MSI project. Click here for file
