# Supplementary material for: The Multiscale Systems Immunology project: software for cell-based immunological simulation
Source: Source Code Biol Med. 2008 Apr 28;3:6. doi: 10.1186/1751-0473-3-6 (PMC2426691; doi:10.1186/1751-0473-3-6)
Supplement: Additional file 1 — MSI source code. Source code for the MSI project. [file 1751-0473-3-6-S1.gz › msi-release-tmp/docs/softmethods/chemotaxis.pdf]

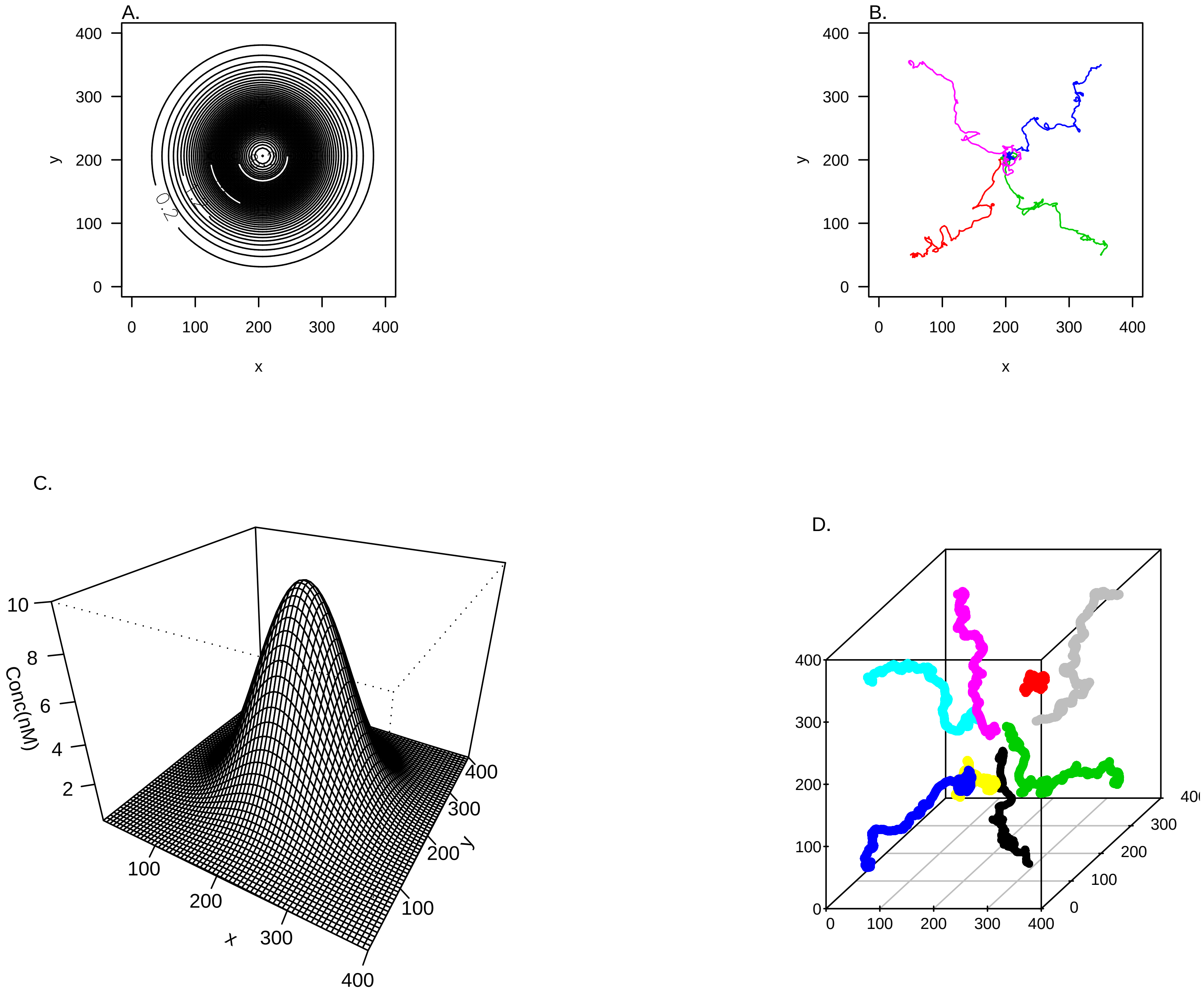
A.
B.
C.
D.
400
300
200
100
0
y
x
0.2
Conc(nM)
10
8
6
4
2
