# Supplementary material for: The Multiscale Systems Immunology project: software for cell-based immunological simulation
Source: Source Code Biol Med. 2008 Apr 28;3:6. doi: 10.1186/1751-0473-3-6 (PMC2426691; doi:10.1186/1751-0473-3-6)
Supplement: Additional file 1 — MSI source code. Source code for the MSI project. [file 1751-0473-3-6-S1.gz › msi-release-tmp/docs/softmethods/response_to_reviewers.doc]

# **Response to Reviewer 1**

**Comments on the software project**

Reviewer 1 had difficulty installing the program on a SUSE Linux distribution. Due to resource constraints, we are currently only supporting binary installations on Debian Etch and Ubuntu Feisty Fawn. However, we have successfully installed and run the MSI software on several other platforms including Mac OS X, Fedora Core, CentOS as well as SUSE. Detailed instructions for installing the MSI software on SUSE 10.2 are given as an appendix to this document.

Reaction-diffusion processes

It is apparent that we have not been clear about just what is spatially extended in our reaction-diffusion integrator. The cells themselves, as cytokine sources, are treated as instantaneously extended, not the cytokine fields. The reason for this is simply that point sources produce singular fields, which makes analysis of numerical error problematic. By considering a smoothly cut-off Gaussian source term, we can state with mathematical certainty that a unique solution exists and that the numerical methods described in the paper will converge to this solution. The most satisfactory approach is to eliminate sources per se altogether and instead impose Neumann boundary conditions on the boundary of each cell to represent the flux of cytokine through the surface. Because the cells are moving, this approach is non-trivial and is under development in our group now. In the meantime, we have found that both point sources and the truncated gaussians described in the paper provide very good approximations outside the cell. Strictly speaking, the domain of the soluble factors should not include the interior of the cell, but the smooth source terms are a good approximation to the secretion of soluble factors from the cell surface. The text has been updated to emphasize that the source terms have compact support, i.e. they are zero outside of the volume of the cell. If the grid size is about the same size as a cell, then there is no difference between point sources and Gaussian sources computationally. The simulation can run with grid sizes that are smaller than the diameter of a cell.

Cellular migration and receptor dynamics

We agree that receptor dynamics plays a key role in the regulation of chemotaxis. As pointed out by the reviewer, the current model for chemotaxis is phenomenological rather than mechanistic, with the response to chemokine concentration and gradient variation “hard wired” directly via Equations (3) and (4). However, it is straightforward to couple the chemotactic response to receptor dynamics, for example, by making the chemotactic parameter Χ a function of some intracellular molecule concentration that is itself regulated by specific receptor signaling. We have not done this yet ourselves because we do not know the detailed parametric knowledge to characterize the link from receptor kinetics and the signal pathway regulating directional motility, but the software is flexible enough to accommodate such a development readily.

Time-splitting for intracellular processes

We would like to clarify that the reaction-diffusion module is only used for soluble factors found extracellularly. Within cells, there is no modeling of explicit spatial structure and hence the reaction-diffusion module is not used. The splitting of the reaction-diffusion-stochastic system is limited to first order due to the stochastic differential equation. Standard second order splitting methods such as Strang splitting will only be first order for this particular model.

**Comments on the report**

Implementation of cell behavior

As suggested by the reviewer, we have added a description of the implementation of cell behavior by passing in behavioral modules as parameters to the cell constructor on Page 6. The caption of Figure 2 has also been considerably expanded to clarify the class plugin concept and relationships among the classes in the Core module.

SQL database description

There are two parts to the construction of a class instance. Instances are created using a generic factory function templated on the class type with the appropriate parameters. These parameters are retrieved from a relational database using SQL given the unique name of the class to be constructed. We have now described this process in more detail on Pages 7-8, as well as the benefits of this approach.

Vessels and cell transport

As described in the manuscript, the current implementation of tissue vasculature simply provides a mechanism for regulating cell immigration rates. These rates can vary depending on the local soluble factor concentrations, which affect the adhesiveness and permeability matrices of the vasculature at that location and hence the probability of cell entry there. Fixing the appropriate matrix entry for permeability to zero can simulate absence of local vasculature. As such, the vasculature does not represent a physical “mini-environment” containing cells and characteristics such as dwell times and directionality within the vasculature are not modeled. However, the reviewer's concept of a structural vessel model is excellent, and we intend to incorporate this suggestion in a future software release.

Spatial dimensions of cells and embedding into space

As far as cell motility is concerned, space is represented as a continuum. Space discretization only comes in when modeling soluble factor concentrations represented as a lattice of points, which is then interpolated to give the local concentration for any given cell location. Cells are represented as elastic 3D spheres, and the collision detection mechanism described in the manuscript determines cell overlap leading to the generation of repulsive forces that result in cells being kept separate.

Intracellular structure

The cells in the MSI software do not have an explicit spatial structure as this would be computationally prohibitive with a large number of cells, and intracellular process modules are typically modeled as linear or nonlinear differential equations coupling aggregate entities representing concentrations of receptors, signaling molecules, transcription factors etc. If necessary, we will implement a spatial approximation using compartmental models to represent (say) the nucleus and cytoplasm, as a trade-off between computational tractability and biological realism.

Step by step walk-through

We have now provided pseudo-code and a walk-through of how a simulation is initialized and run on Pages 14-15, corresponding to the provided sample C++ file *test_simulation.cpp*. At present, it is not possible to run simulations without actually writing code, although it is clear from the example provided in pseudo-code that a GUI can easily be developed to carry out the same steps. Such a user-friendly GUI for simulation specification as well as database population is planned for a future release.

General use of software

We agree that the MSI software is capable of running “generic” cell simulations and have pointed this out on Page 18 as suggested by the reviewer.

Simmune features

We have extended the description of Simmune in the software comparison to highlight the features pointed out by the reviewer.

Model of physiological process

We have previously described the simulation of a physiological model of the regulation of an inflammatory process (REF), and have now provided a simpler but similar example in pseudocode in the manuscript (Page 14-15). We believe that the walk-through, pseudocode and sample code provided make it obvious how to simulate other physiological processes of interest.

**Response to Reviewer 2**

We have cited the software packages CompuCell and Simmune in the Background section as requested.

We apologize for the oversight in missing out Figure citations in the manuscript – this has been corrected and all Figures are now appropriately cited in the text. We have also provided an explanation of Figure 4 in the text on Page 14 and its relevance to the manuscript.

**Appendix – Installation instructions for SuSE 10.2 (from README.suse)**

Instructions for building on Suse.

The following packages can be installed using YaST Some packages are located in additional repositories which can be found using the following website.

http://packages.opensuse-community.org/

You may need a version number greater than or equal to the one suggested below.

The following repositories are suggested below.

http://download.opensuse.org/repositories/devel:/languages:/python/openSUSE_10.2

http://download.opensuse.org/repositories/openSUSE:/10.2/standard

http://download.opensuse.org/repositories/science/openSUSE_10.2

You may need a version number greater than or equal to the one suggested below.

You will also need to download the latest version of SCons 0.97 from www.scons.org. There is an rpm available for download.

To build a working copy of the simulation you will need

Mercurial 0.9.1-24

gcc-c++ 4.1.3-29

gcc-fortran 4.1.3-29

blas 3.1.1-1.1

libblas3 3.1.1-1.1

boost 1.33.1-42

boost-devel 1.33.1-42

python 2.5

python-devel 2.5

python-psycopg2 2.0.6-2.1

python-xml 2.5-19.6

postgresql 8.1.11-0.1

postgresql-devel 8.1.11-0.1

postgresql-libs 8.1.11-0.1

postgresql-server 8.1.11-0.1

libpqxx 2.5.5-33

libpqxx-devel 2.5.5-33

hdf5 1.6.6-11.1

hdf5-devel 1.6.6-11.1

To run tests in the python testing suite you will need

python-gobject 2 2.12.2-11

python-gtk 2.10.3-11

python-gtk-devel 2.10.3-11

python-matplotlib 0.91.2-1.1

python-numarray 1.5.2-2.1 # will work with recommended blas above

python-numpy 1.0.4-3.1 # will work with recommended blas above

python-scipy 0.6.0-3.2 # will work with recommended blas above

For the python vizsualizer you will need

python-imaging 1.1.5-43

python-matplotlib-wx 0.91.2-1.1

python-opengl 1.3-200

python-wxGTK 2.6.3.3-30

You will need to download source for blitz from

http://www.oonumerics.org/blitz

Unpack the tarball and install the library

tar -xzvf blitz-(version #).tar.gz

cd blitz-(version #)

./configure

make install

Special postgresql instructions for Suse.

Once you have installed postgresql you will need to start the postmaster.

Go to the Yast System Service (Runlevel).

Highlight postgresql and select expert. Then check runlevels 3 and 5. If this does not successfully start the postmaster, try

sudo rm -rf /var/lib/pgsql/data

and repeat the previous step.

You will also need to edit some postgresql preferences.

Edit the file /var/lib/pgsql/data/pg_hba.conf so that the last column in the IPv4 and IPv6 local connections says 'md5' instead of 'ident sameuser'

When the above are installed, simply type

$ scons

in the top level directory to compile the code. This invokes the

"scons" build tool to compile the code. This will build libmsi and

test_simulation which are necessary to run a basic simulation.

If you would like to build the unit testing suite and all system

test programs, simply type

$ scons .

The rest of the usage instructions can be found in README under the section title Usage.
