# Supplementary material for: The Multiscale Systems Immunology project: software for cell-based immunological simulation
Source: Source Code Biol Med. 2008 Apr 28;3:6. doi: 10.1186/1751-0473-3-6 (PMC2426691; doi:10.1186/1751-0473-3-6)
Supplement: Additional file 1 — MSI source code. Source code for the MSI project. [file 1751-0473-3-6-S1.gz › msi-release-tmp/docs/softmethods/softmethods.odt]

Building a 3D spatial immune response simulator
FAHEEM MITHA, TIMOTHY LUCAS, FENG FENG, THOMAS B. KEPLER and CLIBURN CHAN, Duke University

INTRODUCTION

Computer simulations are of increasing importance in modeling biological phenomena, complementing both the more traditional mathematical models of mathematical biology and laboratory experiments. While the line between models and simulation is blurred, simulations typically incorporate more biological detail than mathematical models, making it simpler to map the biology directly to the representation at the cost of increased complexity and reduced analytical tractability. Critically, a direct representation allows experimentalists to contribute their expertise in the design, interpretation and critique of in silico experiments, which is crucial for the evolution and improvement of the models. 

Our research interest is in the construction of an individual based immune response simulation that incorporates realistic biophysics and intracellular dynamics, so as to better understand the early immune response to vaccination. We believe that such a framework for immunology simulations can be used by experimentalists to explore the details of molecular and cellular dynamics, much the same way as a new optical technology like two photon microscopy has radically advanced our understanding of leukocyte interactions by allowing direct visualization of in vivo cell motility and interactions. However, the construction of realistic complex spatio-temporal simulations is relatively new to mathematical and theoretical immunology, and poses significant software engineering challenges few researchers in the field will be familiar with. 

The immune response to an antigenic challenge like vaccination involves processes occurring over several temporal and spatial scales, from the intracellular signaling events that occur in individual cells within seconds to minutes, to the complex spatial reorganization that occurs in the draining lymph nodes some distance away a few days later. What happens depends on interactions between several cell types, communicating by direct cell-cell contact and over short distances by diffusible small messenger molecules known as cytokines. The nature of the local environment in which these reactions occur also plays an important role, as there is also cross-talk between the mobile immune cells and resident parenchymal cells. Adding to the complexity, all these interactions may be modulated by the local extracellular matrix, which can buffer and sequester cytokines, forming a local depot which can later be released by specific proteases. While many of these individual interactions have been studied in detail experimentally, there is very little understanding of how these add up to produce a coherent immune response, highlighting the need for a synthetic approach like computer simulation. 

We therefore describe the challenges and trade-offs inherent in building such a simulation, and our specific choices, in the desire that other researchers will have a better understanding of the issues involved and make more informed software engineering choices.

DEVELOPMENT ENVIRONMENT

The first decision was simply the choice of programming language to use. We wanted a design driven by the immunological domain we were simulating, while maintaining the high performance necessary for such a complex simulation, which would have to deal with reaction-diffusion equations as well as the intracellular dynamics and interactions of thousands to millions of immune and parenchymal cells. Our specific needs for a language included support for robust random number libraries, multi-dimensional arrays and parallel computation. While languages like Fortran and C fit these requirements well, they failed at our other requirement of a flexible high-level interface for description of the model domain. C++ includes C as a subset, but also provided object-oriented facilities which would fit our requirements of a high-level interface, and also has the advantage of being well-known and familiar to the scientific community. For better or worse, C++ has strong static typing, and this basic feature to a large extent dictates usage of the language. Strongly typed languages generally have the characteristic that they are both inflexible and have a low level of productivity, particularly in exploratory modes of programming. As a compromise, we opted to use a mixed language environment, where an interpreted dynamically typed language extends a statically typed compiled language. This hybrid system to some extent inherits the advantages of both languages. 

For pragmatic reasons, we decided on a Python/C++ hybrid. Python is a popular object-oriented interpreted language in widespread use as a scripting language, but also has good and ever-improving scientific support. One major reason for the choice of Python is the existence of the Boost Python library, which offers a sophisticated C++ API to automatically convert functions and data types to and from the two languages. One approach to exploiting such a hybrid system is to code most of the simulation in Python, using C++ only for the time-intensive routines. However, we were constrained by the requirement to run the simulation on a Beowulf cluster, and the Python MPI wrappers appear to be unacceptably slow. So we chose to create two functionally identical systems, one in Python and one in C++. Synchrony between the two was enforced by writing a suite of Python unit tests, which depending on a switch, tests either the Python directly or C++ system via Boost Python wrappers. This improved productivity since it is much simpler to code and test a prototype in Python and then port to C++ when we have a working version; than to develop and test a prototype from scratch in C++.

An interesting alternative would have been to code the entire system in a compiled functional language like Objective Caml or Haskell with the Glasgow Haskell Compiler (GHC), which in principle would give the best of both worlds – simple high level design by creating domain specific languages (DSLs) and fast native code. However, as none of the authors had any significant experience with functional languages, this was considered to be too risky for this . We would however, be very interested in hearing about the experiences of other researchers using functional languages in large scale biological simulations.

Just as fundamental were the choices we made for our software development environment. We initially used Subversion for version control, but soon switched to the distributed version control system Mercurial because we found that it was productive to create a new branch to prototype a new idea and merge back once the implementation of the idea was stable, and Mercurial had much better support for this work-flow pattern than Subversion. For software builds, we chose Scons as it allowed us to specify our construction needs and dependencies in Python flexibly. We currently use Roundup for bug and issue management, but are considering moving to Trac as it is appealing to integrate our source code management, bug tracking and documentation with the same web-based package. Not coincidentally, all the tools we chose are Python based, which makes it possible for us to extend these tools if necessary. Choice of IDE or editor is unconstrained, although in practice this tends to be platform specific (Emacs for Linux, Xcode for Macs and Visual Studio for Windows). 

MODULAR DESIGN

One of our design constraints was that the primary domain for our simulation was molecular and cellular immunology, not mathematics, physics or computer science. At the least, this meant that we had to be able to specify and describe a simulation run using the vocabulary of immunology. Another constraint was that we were constantly researching new prototypes, adding more functionality to the system and investigating more efficient data structures and algorithms for performance bottlenecks. So the system had to be extensible; ideally, we could just swap in new functionality for old.

To meet these constraints, we decided on a hierarchical modular system. At the top level, we split the project into Catalog, Experiment and Graphics/Analysis modules. Within each of these were separate sub-modules and classes that actually implemented the functionality of the system. This organization allowed us to develop individual modules independently, so long as there was a standard API that allowed communication between modules. 

The Catalog module is a repository of biological and physical information, for example, detailed parameters governing the motility and division behavior of specific immune cell types, or the diffusion coefficient of a cytokine and the known reactions in which it participates. The information in the Catalog is stored in a postgreSQL relational database, and simulation classes are mapped to database tables using the simplest strategy of mapping each inheritance tree to a table. To facilitate data entry into the Catalog, the Python library SQLAlchemy was used to provide an object-relational mapping, making it possible for developers to create new class specifications without knowing SQL. Eventually, we plan to develop a web interface to the Catalog, probably using Django or Pylons, so that experimentalists and other non-developers can contribute to the population of the database. After the description of the Experiment module, we will describe how the Catalog is used to construct class entities in a simulation at runtime.

Figure 1: Database schema for the Catalog.
The Experiment module is where the code for running a simulation actually resides. To allow the description of an in silico experiment using immunological terminology, there are only a few base classes in the public interface, namely Simulation, Environment, Cell, Soluble_factor and Vessel. Although we are not religious about it, we try to design our classes to have a relatively flat inheritance structure, and to use abstract base classes where possible, as we find that this promotes loose coupling and is easier to maintain.

Simulation is essentially an administrative class, with the ability to run or step through a simulation, log simulation details and also provides hooks to interface with run-time computational steering and visualization. 

The Environment class represents a physical spatial volume, which may be simple when modeling an in vitro environment, or complex when modeling a slice of tissue. Since we do our task allocation in a parallel environment using a spatial decomposition, each processor in a parallel run will contain its own Environment instance. Most of the work done by the Environment class is delegated to three private classes which handle the reaction-diffusion equations (Diffusion), collision detection (Occupation) and cellular immigration and emigration (Traffic). Since these classes are an implementation of the well-known Strategy design pattern, it is simple to upgrade the functionality provided by simply pointing to a new class. For example, we recently swapped in a Multigrid reaction-diffusion algorithm for the original forward Euler scheme with an order of magnitude speedup by simply replacing the Diffusion class in Environment. The Environment also serves as a container for blood and lymphatic vessels (Vessel), various cell types (Cell), and various cytokines and chemokines (Soluble_factor), and manages the interactions among these components. 

Similar to Environment, the Cell base class is also a composite class, with distinct behaviors delegated to their own classes, and hence also easily extensible. In a real cell, the functional state is determined by the locations and local concentrations of various proteins, as well as various chemical modifications to the proteins (e.g. phosphorylation). These characteristics of a protein are themselves dynamically regulated, and typically modeled using nonlinear ordinary differential equations. Importantly these proteins determine the functional behavior of the cell, and it is sufficient to couple cell behavior modules to these protein concentrations to model cell stimulus-response behavior. The protein readouts are encapsulated in a State class, which tracks and exposes the fluctuating protein levels. In our current implementation, there is a mechanistic model that governs the protein levels, in which an explicit cascade with models for Receptor, Inducer, Promoter, Gene and other Proteins govern the level of any given protein. However, it would be simple to replace this mechanistic model with a simpler probabilistic model, or even a much more detailed mechanistic model depending on the goals of the simulation. By having a flexible granularity, we can study an immune response at different resolutions – for example, when studying the behavior of individual cells, we can turn up the resolution, while for studying large population behaviors, we would probably use a coarser approximation of internal cell dynamics. Other behaviors like Motility and Secretion can then base the quality/quantity of their response at any time step on the internal environment represented by the State class and/or the external environment (e.g. concentration or gradient of various cytokines or cell-cell contacts). This allows an extremely flexible stimulus-response coupling between the internal and external cellular environments and cellular behavior. Because of the loose coupling and the ability to change cellular behavior by “plug-in”, we do not actually find it necessary to have an inheritance hierarchy to represent the different cell types. Instead, we just “plug-in” different behavior modules for the various cell types to simulate their observed behavior. 

The Vessel class represents blood vessels which permeate most tissues densely. Immune cells typically enter tissues by migrating across the endothelial barrier at blood capillaries, and egress tissues at post-capillary venules to enter the lymphatic system. The rate of entry and egress of various cell types is regulated by the presence or absence of inflammatory cytokines which regulate the adhesiveness and permeability of the blood vessels, as well as the presence or absence of chemokines which attract immune cells. Our implementation of blood vessels is simple – the entry and egress vessels are represented by matrices representing positions in space, and the probability of cell trafficking at a point depends on the permeability and adhesiveness of the corresponding matrix entry, which in turn is sensitive to the local concentration of the relevant soluble factors. We currently assume that the blood vessels are homogeneously distributed, it is trivial to alter this to reflect tissue histology more accurately by masking certain matrix entries.

The Soluble_factor class is essentially a container for a matrix of concentration, with a few accessor and mutator methods for convenience and some basic attributes including its diffusion coefficient. An instantiation of the Soluble_factor class is made for every cytokine and chemokine we are interested in modeling, and the matrix is updated by the Diffusion class which takes into account secretion from cell sources, absorption by cell or extracellular matrix sinks, reaction between soluble factors and diffusion.

Figure 2: Class composites and aggregates in the Experiment module.

While the bundling of distinct behaviors into separate classes certainly makes it simpler to configure classes dynamically, instantiation of actual instances can be clumsy, accounting for a significant proportion of the code required to run a simulation. However, biological cells, once adequately characterized, can be reused in many different simulations, just like an experimentalist would use the same cell lines (possibly ordered from a catalog!) for many different experiments. This was the idea driving the creation of the Catalog module, and here we will describe how it is used for instantiation of Experiment classes.

We started by writing generic code that could construct an class instance without knowing in advance the type of the instance or even its constructor parameters. This would provide the ﬂexibility to easily incorporate new classes in the database, or new subclasses of currently existing types. To do so, we adopted the object factories scheme [Alexandrescu:2001], which allows ﬂexible creation of object instances using C++ templates. We also extended the factory to take arbitrary constructor parameters with partial template specialization, and wrote a Python script to generate templates for up to 20 arbitrary constructor arguments. To create a factory for a particular class, we instantiate the factory and register it with the appropriate constructor arguments passed in as a string. 
While this process is a little involved, it only has to be done once for each class, and the typical user does not have to be exposed to any of it, since all that needs to be done once the infrastructure is in place is to call a function like create cell(”macrophage”) which will call the appropriate constructor for a macrophage. In practice, we also need to connect to a local or remote database containing the data, but this is easily done with the libpqxx C++ API for postgreSQL at the beginning of the simulation.

To visualize a running system, we embed a lightweight web server associated with each processor that allows us to peek at and potentially alter simulation instance variables on the fly. This computational steering system has a GUI frontend and is designed in the Model-View-Controller paradigm, allowing for flexible configuration. For offline visualization and analysis, we export simulation variables using the standard HDF5 format, which can then be visualized using our own wxPython and VTK based visualization application or imported into a statistical package like R or Matlab for further analysis.

Fig 3. Visualization of a 400 by 400 by 400 mm simulation with 3 chemokine sources showing the tracks of immigrating cells taken at 10 minute intervals. Different colors reflect differential activation of the genes for tumor necrosis factor (TNF) and soluble TNF receptor regulator proteins, which are activated stochastically in response to the local chemokine concentration.

TESTING

We run unit tests using the Python unittest module, with the only novelty being that since all our C++ classes are compiled to a shared library readable in Python, we can drive both Python and C++ unit tests with the same test code. At a slightly higher level, we also compare simulated behavior with analytical predictions where closed form solutions (either deterministic or statistical) are available, for example, in testing cell motility modeled as Langevin processes, or simple diffusion from a single source secreting at a constant rate. There are also informal “eyeball” tests, in which simulations with simple configurations are run and visually checked to ensure that the resulting behavior is consistent with experimental results. We have not incorporated code coverage analysis or continuous build systems yet, but these would certainly be sensible additions. 

OPTIMIZATION

Due to the highly modular nature of our code, it is generally simple to replace an inefficient module with a more efficient one. Identification of bottlenecks is done using the callgrind tool widely available on Unix platforms, and the kcachegrind GUI to visualize the resulting profile. Examples of such high level optimizations include the replacement of a forward Euler diffusion scheme with a Multigrid scheme, and replacing collision detection by looping over all cells with a grid-based method  implemented as a hash table. At a lower level, we have removed unnecessary copies by using references where possible for large data structures, and rearranged code to minimize unnecessary looping. So far we have avoided doing micro-optimizations (e.g. loop unrolling) believing that such tasks are best left to the compiler. A particular simple optimization was to specify the appropriate compiler flags for each machine architecture, allowing the compiler to make optimal use of pipelining and vectorization.

ROADMAP

The code is currently functional and we are using it to run experimental simulations. Source code, documentation and a community wiki can be found at Multiscale Systems Immunology website (http://xxx.xxx.xxx), and typical usage examples have been published in a recent review [IR:2007]. In the near future, our priorities are to populate the Catalog with more cell types, soluble factors and types of environments, so that more realistic and complex immune simulations can be conducted. We also need to fully parallelize the code using MPI so that larger scale simulations can be run in a reasonable time on a Beowulf cluster. This should not pose too much difficulty since we have a prototype that already works in parallel; we just need to incorporate recently added features of the simulation to this prototype. Finally, we would like to simulate heterogeneous tissue environments with the architecture specified from real tissue histology – this would require a new PDE engine that can handle heterogeneous boundaries efficiently and is being actively researched by our group.

CONCLUSION

There are many software engineering techniques that can make it more pleasant (or less painful) and productive to develop a large and complex scientific simulation. However, since most modelers come from a more theoretical background and coding experience is limited to smaller one person efforts, these may not be familiar to researchers in computational biology. We have described some of the tools and methods that we have found useful in the construction of an individual based immune simulation, including setting up a sensible version control, build system and testing suites, the use and integration of a relational database, modular decomposition of the project, object-oriented design incorporating ideas from domain-driven design and the patterns community, and the use of standard formats to facilitate communication between subsystems (RDBMS, HDF5). Rather unusually, we have also opted to use a mixed language approach to software development - this has worked well for us, and we would encourage other simulation groups to give it a try. Hopefully, this essay will help other groups develop better and more sophisticated simulators for molecular and cellular biology, and allow us to reach a more holistic and integrated understanding of the organism.
