# Supplementary material for: The Multiscale Systems Immunology project: software for cell-based immunological simulation
Source: Source Code Biol Med. 2008 Apr 28;3:6. doi: 10.1186/1751-0473-3-6 (PMC2426691; doi:10.1186/1751-0473-3-6)
Supplement: Additional file 1 — MSI source code. Source code for the MSI project. [file 1751-0473-3-6-S1.gz › msi-release-tmp/docs/softmethods/catalog.pdf]

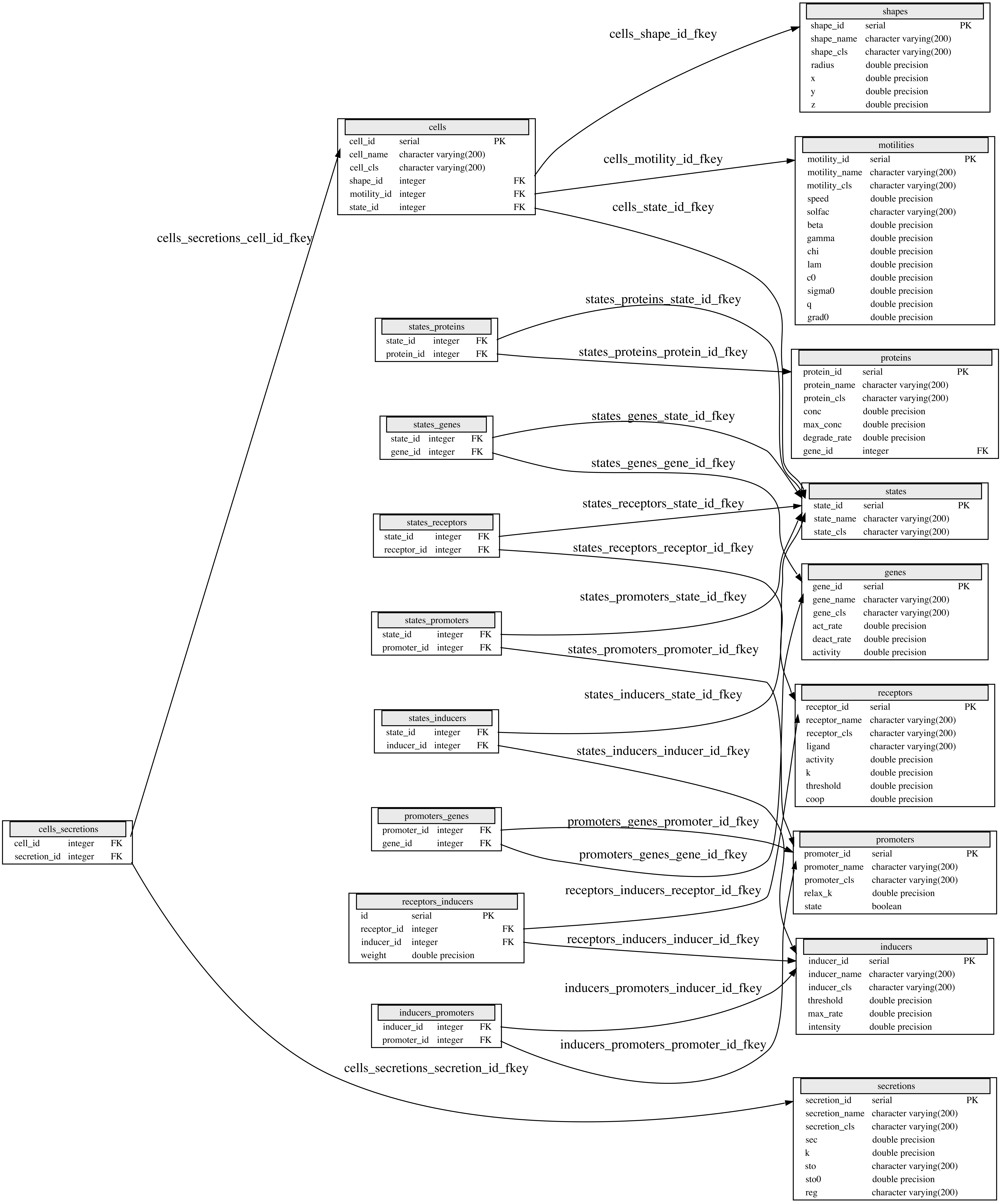
shapes
shape_id serial PK
shape_name character varying(200)
shape_cls character varying(200)
radius double precision
x double precision
y double precision
z double precision
cells_shape_id_fkey
cells
cell_id serial PK
cell_name character varying(200)
cell_cls character varying(200)
shape_id integer FK
motility_id integer FK
state_id integer FK
cells_motility_id_fkey
motilities
motility_id serial PK
motility_name character varying(200)
motility_cls character varying(200)
speed double precision
solfac character varying(200)
beta double precision
gamma double precision
chi double precision
lam double precision
c0 double precision
sigma0 double precision
q double precision
grad0 double precision
cells_state_id_fkey
cells_secretions_cell_id_fkey
states_proteins_state_id_fkey
states_proteins
state_id integer FK
protein_id integer FK
states_proteins_protein_id_fkey
proteins
protein_id serial PK
protein_name character varying(200)
protein_cls character varying(200)
conc double precision
max_conc double precision
degrade_rate double precision
gene_id integer FK
states_genes_state_id_fkey
states_genes
state_id integer FK
gene_id integer FK
states_genes_gene_id_fkey
states
state_id serial PK
state_name character varying(200)
state_cls character varying(200)
states_receptors_state_id_fkey
states_receptors
state_id integer FK
receptor_id integer FK
states_receptors_receptor_id_fkey
genes
gene_id serial PK
gene_name character varying(200)
gene_cls character varying(200)
act_rate double precision
deact_rate double precision
activity double precision
states_promoters_state_id_fkey
states_promoters
state_id integer FK
promoter_id integer FK
states_promoters_promoter_id_fkey
states_inducers_state_id_fkey
states_inducers
state_id integer FK
inducer_id integer FK
receptors
receptor_id serial PK
receptor_name character varying(200)
receptor_cls character varying(200)
ligand character varying(200)
activity double precision
k double precision
threshold double precision
coop double precision
states_inducers_inducer_id_fkey
cells_secretions
cell_id integer FK
secretion_id integer FK
promoters_genes
promoter_id integer FK
gene_id integer FK
promoters_genes_promoter_id_fkey
promoters_genes_gene_id_fkey
promoters
promoter_id serial PK
promoter_name character varying(200)
promoter_cls character varying(200)
relax_k double precision
state boolean
receptors_inducers_receptor_id_fkey
receptors_inducers
id serial PK
receptor_id integer FK
inducer_id integer FK
weight double precision
receptors_inducers_inducer_id_fkey
inducers
inducer_id serial PK
inducer_name character varying(200)
inducer_cls character varying(200)
threshold double precision
max_rate double precision
intensity double precision
inducers_promoters_inducer_id_fkey
inducers_promoters
inducer_id integer FK
promoter_id integer FK
inducers_promoters_promoter_id_fkey
cells_secretions_secretion_id_fkey
secretions
secretion_id serial PK
secretion_name character varying(200)
secretion_cls character varying(200)
sec double precision
k double precision
sto character varying(200)
sto0 double precision
reg character varying(200)
