# Supplementary material for: The Multiscale Systems Immunology project: software for cell-based immunological simulation
Source: Source Code Biol Med. 2008 Apr 28;3:6. doi: 10.1186/1751-0473-3-6 (PMC2426691; doi:10.1186/1751-0473-3-6)
Supplement: Additional file 1 — MSI source code. Source code for the MSI project. [file 1751-0473-3-6-S1.gz › msi-release-tmp/docs/softmethods/classes.pdf]

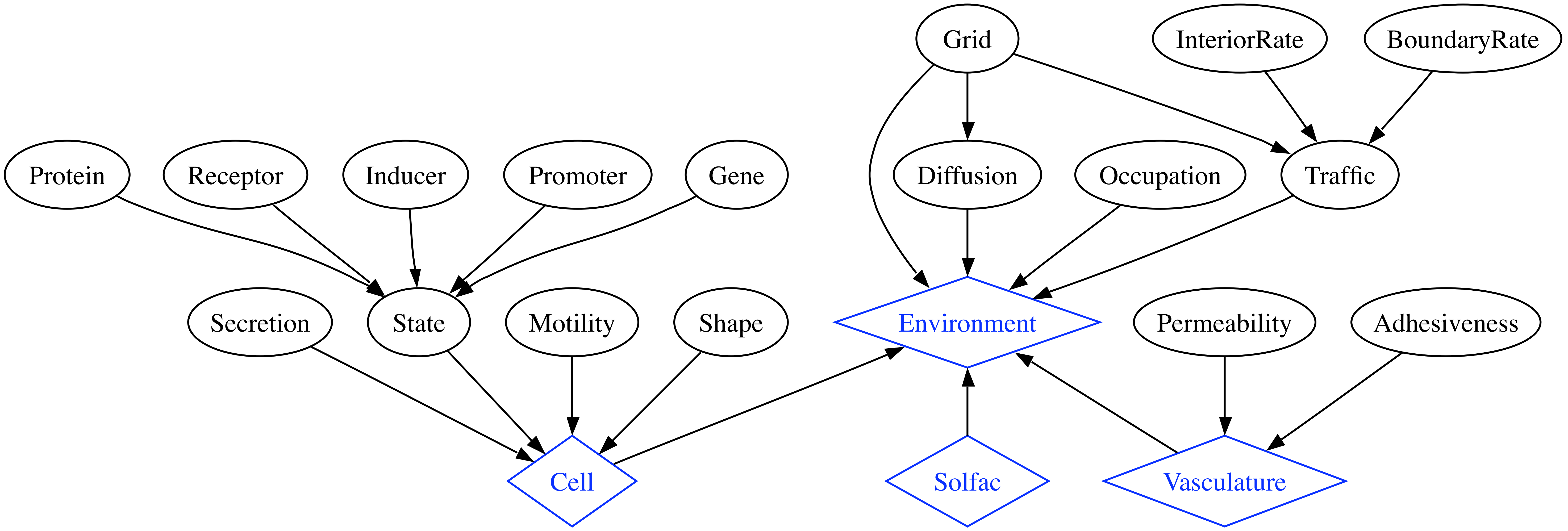
Grid
InteriorRate
BoundaryRate
Protein
Receptor
Inducer
Promoter
Gene
Diffusion
Occupation
Traffic
Secretion
State
Motility
Shape
Environment
Permeability
Adhesiveness
Cell
Solfac
Vasculature
