# Supplementary material for: The Multiscale Systems Immunology project: software for cell-based immunological simulation
Source: Source Code Biol Med. 2008 Apr 28;3:6. doi: 10.1186/1751-0473-3-6 (PMC2426691; doi:10.1186/1751-0473-3-6)
Supplement: Additional file 1 — MSI source code. Source code for the MSI project. [file 1751-0473-3-6-S1.gz › msi-release-tmp/docs/softmethods/typeinst.pdf]

Lecture Notes in Computer Science: Authors' Instructions for the Preparation of Camera-Ready Contributions to LNCS/LNAI/LNBI Proceedings

Alfred Hofmann, Ursula Barth, Ingrid Beyer, Christine Günther, Frank Holzwarth, Anna Kramer, and Erika Siebert-Cole

Springer-Verlag, Computer Science Editorial,
Tiergartenstr. 17, 69121 Heidelberg, Germany
{alfred.hofmann,ursula.barth,ingrid.beyer,christine.guenther, frank.holzwarth,anna.kramer,erika.siebert-cole,lncs}@springer.com
http://www.springer.com/lncs

Abstract. The abstract should summarize the contents of the paper and should contain at least 70 and at most 150 words. It should be written using the *abstract* environment.

Key words: We would like to encourage you to list your keywords within the abstract section

1 Introduction

You are strongly encouraged to use LaTeX 2_ε for the preparation of your camera-ready manuscript together with the corresponding Springer class file `llncs.cls`. Only if you use LaTeX 2_ε can hyperlinks be generated in the online version of your manuscript.

The LaTeX source of this instruction file for LaTeX users may be used as a template. Kindly send the final and checked source and PDF files of your paper to the Contact Volume Editor. This is usually one of the organizers of the conference. You should make sure that the LaTeX and the PDF files are identical and correct and that only one version of your paper is sent. It is not possible to update files at a later stage. Please note that we do not need the printed paper.

1.1 Checking the PDF File

Kindly assure that the Contact Volume Editor is given the name and email address of the contact author for your paper. The Contact Volume Editor uses these details to compile a list for our production department at SPS in India. Once the files have been worked upon, SPS sends a copy of the final pdf of each paper to its contact author. The contact author is asked to check through the final pdf to make sure that no errors have crept in during the transfer or

preparation of the files. This should not be seen as an opportunity to update or copyedit the papers, which is not possible due to time constraints. Only errors introduced during the preparation of the files will be corrected.

This round of checking takes place about two weeks after the files have been sent to the Editorial by the Contact Volume Editor, i.e. roughly seven weeks before the start of the conference for conference proceedings, or seven weeks before the volume leaves the printer's, for post-proceedings. If SPS does not receive a reply from a particular contact author, within the timeframe given, then it is presumed that the author has found no errors in the paper. The tight publication schedule of LNCS does not allow SPS to send reminders or search for alternative email addresses on the Internet.

In some cases, it is the Contact Volume Editor that checks all the final pdfs. In such cases, the authors are not involved in the checking phase.

1.2 Additional Information Required by the Volume Editor

When sending your final files, please include a readme informing the Contact Volume Editor which of your names is/are your first name(s) and which is/are your family name(s). This is particularly important for Spanish and Chinese names. Authors are listed alphabetically according to their surnames in the author index.

1.3 Copyright Forms

The copyright form may be downloaded from the "For Authors" (Information for LNCS Authors) section of the LNCS Website: `www.springer.com/lncs`. Please send your signed copyright form to the Contact Volume Editor, either as a scanned pdf or by fax or by courier. One author may sign on behalf of all the other authors of a particular paper. Digital signatures are acceptable.

2 Paper Preparation

Springer provides you with a complete integrated LaTeX document class (`llncs.cls`) for multi-author books such as those in the LNCS series. Papers not complying with the LNCS style will be reformatted. This can lead to an increase in the overall number of pages. We would therefore urge you not to squash your paper.

Please always cancel any superfluous definitions that are not actually used in your text. If you do not, these may conflict with the definitions of the macro package, causing changes in the structure of the text and leading to numerous mistakes in the proofs.

If you wonder what LaTeX is and where it can be obtained, see the "*LaTeX project site*" (`http://www.latex-project.org`) and especially the webpage "*How to get it*" (`http://www.latex-project.org/ftp.html`) respectively.

When you use LaTeX together with our document class file, `llncs.cls`, your text is typeset automatically in Computer Modern Roman (CM) fonts. Please

do *not* change the preset fonts. If you have to use fonts other than the preset fonts, kindly submit these with your files.

Please use the commands `\label` and `\ref` for cross-references and the commands `\bibitem` and `\cite` for references to the bibliography, to enable us to create hyperlinks at these places.

For preparing your figures electronically and integrating them into your source file we recommend using the standard LaTeX `graphics` or `graphicx` package. These provide the `\includegraphics` command. In general, please refrain from using the `\special` command.

Remember to submit any further style files and fonts you have used together with your source files.

Headings. Headings should be capitalized (i.e., nouns, verbs, and all other words except articles, prepositions, and conjunctions should be set with an initial capital) and should, with the exception of the title, be aligned to the left. Words joined by a hyphen are subject to a special rule. If the first word can stand alone, the second word should be capitalized.

Here are some examples of headings: "Criteria to Disprove Context-Freeness of Collage Language", "On Correcting the Intrusion of Tracing Non-deterministic Programs by Software", "A User-Friendly and Extendable Data Distribution System", "Multi-flip Networks: Parallelizing GenSAT", "Self-determinations of Man".

Lemmas, Propositions, and Theorems. The numbers accorded to lemmas, propositions, and theorems, etc. should appear in consecutive order, starting with Lemma 1, and not, for example, with Lemma 11.

2.1 Figures

For LaTeX users, we recommend using the *graphics* or *graphicx* package and the `\includegraphics` command.

Please check that the lines in line drawings are not interrupted and are of a constant width. Grids and details within the figures must be clearly legible and may not be written one on top of the other. Line drawings should have a resolution of at least 800 dpi (preferably 1200 dpi). The lettering in figures should have a height of 2 mm (10-point type). Figures should be numbered and should have a caption which should always be positioned *under* the figures, in contrast to the caption belonging to a table, which should always appear *above* the table; this is simply achieved as matter of sequence in your source.

Please center the figures or your tabular material by using the `\centering` declaration. Short captions are centered by default between the margins and typeset in 9-point type (Fig. 1 shows an example). The distance between text and figure is preset to be about 8 mm, the distance between figure and caption about 6 mm.

To ensure that the reproduction of your illustrations is of a reasonable quality, we advise against the use of shading. The contrast should be as pronounced as possible.

If screenshots are necessary, please make sure that you are happy with the print quality before you send the files.

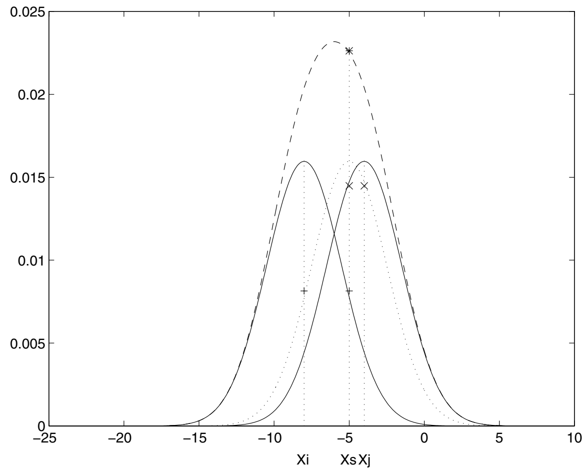

Fig. 1. One kernel at x_s (*dotted kernel*) or two kernels at x_i and x_j (*left and right*) lead to the same summed estimate at x_s. This shows a figure consisting of different types of lines. Elements of the figure described in the caption should be set in italics, in parentheses, as shown in this sample caption.

Please define figures (and tables) as floating objects. Please avoid using optional location parameters like "`[h]`" for "here".

Remark 1. In the printed volumes, illustrations are generally black and white (halftones), and only in exceptional cases, and if the author is prepared to cover the extra cost for color reproduction, are colored pictures accepted. Colored pictures are welcome in the electronic version free of charge. If you send colored figures that are to be printed in black and white, please make sure that they really are legible in black and white. Some colors as well as the contrast of converted colors show up very poorly when printed in black and white.

2.2 Formulas

Displayed equations or formulas are centered and set on a separate line (with an extra line or halfline space above and below). Displayed expressions should be numbered for reference. The numbers should be consecutive within each section or within the contribution, with numbers enclosed in parentheses and set on the right margin – which is the default if you use the *equation* environment, e.g.

$$\psi(u) = \int_o^T \left[\frac{1}{2} \left(\Lambda_o^{-1} u, u \right) + N^*(-u) \right] dt \; . \tag{1}$$

Please punctuate a displayed equation in the same way as ordinary text but with a small space before the end punctuation.

2.3 Footnotes

The superscript numeral used to refer to a footnote appears in the text either directly after the word to be discussed or – in relation to a phrase or a sentence – following the punctuation sign (comma, semicolon, or period). Footnotes should appear at the bottom of the normal text area, with a line of about 2 cm set immediately above them.[1]

2.4 Program Code

Program listings or program commands in the text are normally set in typewriter font, e.g., CMTT10 or Courier.

Example of a Computer Program

```
program Inflation (Output)
  {Assuming annual inflation rates of 7%, 8%, and 10%,...
   years};
   const
     MaxYears = 10;
   var
     Year: 0..MaxYears;
     Factor1, Factor2, Factor3: Real;
   begin
     Year := 0;
     Factor1 := 1.0; Factor2 := 1.0; Factor3 := 1.0;
     WriteLn('Year  7% 8% 10%'); WriteLn;
     repeat
       Year := Year + 1;
       Factor1 := Factor1 * 1.07;
       Factor2 := Factor2 * 1.08;
       Factor3 := Factor3 * 1.10;
       WriteLn(Year:5,Factor1:7:3,Factor2:7:3,Factor3:7:3)
     until Year = MaxYears
end.
```

(Example from Jensen K., Wirth N. (1991) Pascal user manual and report. Springer, New York)

[1] The footnote numeral is set flush left and the text follows with the usual word spacing.

2.5 Citations

The list of references is headed "References" and is not assigned a number. The list should be set in small print and placed at the end of your contribution, in front of the appendix, if one exists. Please do not insert a pagebreak before the list of references if the page is not completely filled. An example is given at the end of this information sheet. For citations in the text please use square brackets and consecutive numbers: [1], [2], [3] ...

2.6 Page Numbering and Running Heads

There is no need to include page numbers. If your paper title is too long to serve as a running head, it will be shortened. Your suggestion as to how to shorten it would be most welcome.

3 LNCS Online

The online version of the volume will be available in LNCS Online. Members of institutes subscribing to the Lecture Notes in Computer Science series have access to all the pdfs of all the online publications. Non-subscribers can only read as far as the abstracts. If they try to go beyond this point, they are automatically asked, whether they would like to order the pdf, and are given instructions as to how to do so.

4 BibTeX Entries

The correct BibTeX entries for the Lecture Notes in Computer Science volumes can be found at the following Website shortly after the publication of the book: `http://www.informatik.uni-trier.de/~ley/db/journals/lncs.html`

Acknowledgments. The heading should be treated as a subsubsection heading and should not be assigned a number.

References

1. van Leeuwen, J. (ed.): Computer Science Today. Recent Trends and Developments. Lecture Notes in Computer Science, Vol. 1000. Springer-Verlag, Berlin Heidelberg New York (1995)
2. Bruce, K.B., Cardelli, L., Pierce, B.C.: Comparing Object Encodings. In: Abadi, M., Ito, T. (eds.): Theoretical Aspects of Computer Software. Lecture Notes in Computer Science, Vol. 1281. Springer-Verlag, Berlin Heidelberg New York (1997) 415–438
3. Michalewicz, Z.: Genetic Algorithms + Data Structures = Evolution Programs. 3rd edn. Springer-Verlag, Berlin Heidelberg New York (1996)
4. Baldonado, M., Chang, C.-C.K., Gravano, L., Paepcke, A.: The Stanford Digital Library Metadata Architecture. Int. J. Digit. Libr. **1** (1997) 108–121

Appendix: Springer-Author Discount

LNCS authors are entitled to a 33.3% discount off all Springer publications. Before placing an order, the author should send an email to `SDC.bookorder@springer.com`, giving full details of his or her Springer publication, to obtain a so-called token. This token is a number, which must be entered when placing an order via the Internet, in order to obtain the discount.

5 Checklist of Items to be Sent to Volume Editors

Here is a checklist of everything the volume editor requires from you:

- ☐ The final LaTeX source files
- ☐ A final PDF file
- ☐ A copyright form, signed by one author on behalf of all the authors of the paper.
- ☐ A readme giving the first name(s) and the surname(s) of all the authors of the paper, as well as the name and address of the corresponding author.
